# The Psychopathology of Ink: Tattoos as a Window into Personality (Disorders)

**DOI:** 10.1192/j.eurpsy.2025.1920

**Published:** 2025-08-26

**Authors:** T. Grahovac Juretić, A. Mančić, E. Dadić-Hero, M. Letica-Crepulja, K. Ružić

**Affiliations:** 1 Department of psychiatry, University Hospital Centre Rijeka, Rijeka, Croatia

## Abstract

**Introduction:**

Tattoos, once viewed primarily as symbols of rebellion or cultural affiliation, have become increasingly prevalent and socially accepted across diverse populations. This shift raises questions about the psychological motivations behind body art, particularly in individuals with personality disorders.

**Objectives:**

These case studies explore the potential significance of tattoos in the context of personality psychopathology, examining how tattoos may serve as externalized representations of inner conflicts, identity fragmentation, and unmet emotional needs.

**Methods:**

Through the lens of theory, we will discuss how tattoos can function as a form of self-expression and self-regulation, offering insight into defense mechanisms such as splitting, projection, and sublimation in individuals with borderline, narcissistic, and antisocial personality disorders.

**Results:**

The presentation will also explore the therapeutic implications of tattoos, considering their potential as entry points for understanding the symbolic and emotional worlds of patients with personality disorders. We will also reflect on the clinician’s role in addressing tattoos in psychotherapy, balancing sensitivity with inquiry, and understanding how body art may influence the therapeutic alliance.

**Conclusions:**

Through the session we aim to deepen our understanding of tattoos as meaningful psychological markers in modern psychiatric practice.

**Disclosure of Interest:**

None Declared

